# Domestic Summary

**Published:** 1838-12-19

**Authors:** 


### DOMESTIC SUMMARY.

Dr. Charles Bell Gibson has been appointed one of the Accoucheurs to the Philadelphia Hospital.

Dr. Thomas Smith, of Darlington District, has been elected Lieutenant Governor of the State of South Carolina.

In the Southern Medical and Surgical Journal for November, Dr. R. D. Arnold reports an interesting case of *Rupture of the Rectum and protrusion of the Intestines through it, and their expulsion externally per anum*, which occurred in the practice of Dr. G. C. Habersham. During life, the whole small intestine and a portion of the colon had protruded from the anus. Dissection revealed the extraordinary fact of a *rupture of the rectum* upon its anterior portion, five inches above the anus, through which aperture the intestines had passed: there was no rupture of the sphincter ani.

We have received and read with pleasure a well-written and sound discourse on the *True Value of Experience in Medicine*—an introductory lecture by Prof. H. Miller, of the Louisville Medical Institute.

*Reduction of a Dislocated Shoulder of seventy-two days' standing.*—The Boston Medical and Surgical Journal for November 28th, contains an account of a successful operation for a luxation of the humerus downwards, of seventy-two days' standing, by Dr. Flint, of Northampton. The head of the bone was closely adherent to the side, so closely that it was impossible to carry out the arm, for the shortest distance, without exquisite pain. The motions of the arm were very much embarrassed, and the use of the fingers and hand almost entirely lost. Failing in effecting the reduction by the usual means, "it occurred to Dr. Flint, that nature had probably filled up the socket, and that the cavity might be cleared for receiving the bone, by brisk rotation after the requisite extension was made." Under this process the reduction was accomplished at a moment of complete syncope. But little inflammation followed, and in six months the use of the arm was nearly regained.

*Works announced.*—A new edition of Dr. Forbe's translation of Laennec on the Chest, with Andral's Notes, and Remarks on Cerebral Auscultation, by J. D. Fisher, of Boston.—A translation of Billard's Treatise on Infantile Diseases, by Dr. James Stewart, of New York.—A Treatise on the Theory and Practice of Medicine, by Joseph A. Gallup, M. D., formerly professor of that branch in the Castleton Medical School.

*Medical Prize.*—The Medical Society of Augusta, Ga., offer a Prize of Fifty Dollars, or its equivalent, to be designated by the successful competitor, for the best *approved* original Essay *on the use and abuse of Calomel, as a therapeutic agent.*

The following are the arrangements adopted by the society:

1. The essay shall not exceed 40 octavo pages.

2. Essays, intended for the competition, are to be directed, *free of expense*, "To the Secretary of the Medical Society of Augusta, Ga.," and must be in his possession by the 1st day of May, 1839. Each essay must be endorsed with a *motto*, which must be also on an accompanying sealed letter, containing the name and address of the writer.

3. The Medical Society will, as a body, proceed to the reading and inspection of all the essays received by the Secretary, as soon as practicable after the 1st day of May, 1839, and will determine, by the vote of the majority, on the successful essay. After such decision, the letter bearing the corresponding *motto*, will be opened, and the essay published under the name of the author, in the Southern Medical and Surgical Journal.

4. Should none of the essays be judged worthy of the prize proposed, they will remain in the hands of the secretary, subject to the order of their authors, for three months, the names remaining under seal; after which, if not otherwise directed, they will be considered the property of the Society.

*Extirpation of the Parotid Gland.* By N. R. Smith, M. D.—August 21, 1835, my friend, Dr. T. E. Bond, sen., requested me to examine a tumour on the face of Miss Bryan, the daughter of Charles Bryan, of Baltimore county. It was located between the left ear and the angle of the jaw, in the precise situation of the parotid gland. It presented an abrupt eminence, something in form like a pointed phlegmon—its base not broad. The tumour was hard, occasionally affected with lancinating pains, and tender to the touch. It very much disfigured the patient, and was stated to be increasing in a degree which caused much anxiety.

Both myself and Dr. Bond were inclined to regard the tumour as one which had originated in a lymphatic ganglion lying on the parotid, although we could not well define its base and extent in the direction of the zygomatic fossa. The history of the case, in our view, justified an operation for the removal of the part diseased, and I accordingly undertook it.

In the presence of several of my pupils, August 25, 1836, I commenced the operation by making a vertical incision from the zygoma to the angle of the jaw; and, deepening it, laid bare the external aspect of the tumour. On endeavouring to define its lateral limits with the knife, I soon discovered that I had to deal with the entire parotid, and proceeded accordingly. The disease affecting the organ had, in regard to consistence and form, distinguished itself from the surrounding parts. It was much more globular than the healthy gland—had a more distinct envelope of cellular tissue, and had receded in a degree from its confined situation. Penetrating the posterior part of the tumour near its surface, I soon traced out the facial nerve (portio dura) and separated it from the tumour to a considerable extent. I then doubted whether to attempt to disengage the tumour from beneath the nerve, or to divide the latter. Anticipating great embarrassment in the execution of the first plan, and fearing that serious irritation would be necessarily inflicted upon the nerve, I at once divided it. Paralysis of the muscles of the face on that side instantly resulted.

I then cautiously proceeded with the dissection of the tumour. Penetrating between its diseased lobules on every side were occasional bands of cellular tissue. These I divided with great caution, as I expected to find some of them involving the branches of the external carotid, which emanate from the parotid. Thrusting the index of my left hand beneath the tumour, I made them successively turn over its extremity; and carefully feeling for pulsation, I effected their division, sometimes with the knife, but more generally with a very narrow probe-pointed bistoury. When I felt pulsation I endeavoured to effect the laceration of the band with the finger, or the handle of a scalpel. Thus I proceeded till I had insulated the tumour with the exception of a single band attaching the upper and posterior part of the diseased mass to the deep temporal region. Occasionally there had sprung a small artery, but not furnishing sufficient blood to embarrass the operation. I now divided the last band which attached the tumour, and a single artery sprung with considerable impetuosity. This I secured without difficulty with the tenaculum. Had I felt any considerable pulsation in it, I should have included the whole band in a ligature before effecting its separation.

The tumour being now removed, I explored the cavity from which it had been taken. This extended quite to the styloid process, and the muscles arising from that point were seen with perfect distinctness. Not a vestige of any thing presenting the appearance of the parotid gland could be seen in the space usually occupied by it. Probably, however, that small process of the gland which extends forward on the cheek, termed socia parotidis, was left; but in the incision along the anterior border of the tumour I did not distinguish it or the duct of Steno. The tumour is in my possession, and is of the size of a very large hickory nut.

The patient bore the operation with much fortitude. The wound was dressed lightly with lint and bandage. Inflammation to some extent arose, and some embarrassment of deglutition and respiration resulted. A common cataplasm was applied; and, on the occurrence of suppuration, the unpleasant symptoms abated. Cicatrization was effected in a few days, and all morbid sufferance ceased. The face, however, remained paralysed; and the eye suffered in some degree from the inability of the patient to close it. I saw this young lady some months after her recovery; and, at that time, the cicatrix remained healthy, and the paralysis of the face had decidedly diminished.

The extirpation of the gland in this case I accomplished with much greater ease than I had expected. The small amount of hæmorrhage cannot fail to strike the reader with some surprise. It is to be accounted for, in my opinion, by presuming the obliteration of many of the vessels by the enlargement and morbid hardness of the glands; also by its displacement, in consequence of which traction was made on the vessels issuing from it.

This case furnishes facts which will aid to reconcile the opinions of anatomists and surgeons relative to the feasibility of the extirpation of this gland. The former, even at the present day, observing the extreme difficulty of dissecting the healthy gland, often declare its removal impossible. Surgeons, however, report numerous cases in which the operation has been unquestionably performed. I have seen the operation performed with success by Professor M'Clellan, of Philadelphia; and in that case also the hæmorrhage was trifling, and from but a single vessel. The gland has also been removed by the late Professor Davidge of this city—by the late Professor N. Smith—by Professor Dudley, and, I believe, by several others in this country, as well as by numerous surgeons abroad.

The feasibility of the operation in these cases is, in my opinion, to be explained by the facts furnished in the above instance. The tumour in its growth had assumed a harder consistence than natural, without having imparted disease to the surrounding parts. It was therefore better defined than the healthy organ. It had also become spheroidal; and, from its size and hardness, had necessarily receded from its confined situation. Its extirpation was therefore undoubtedly far easier than would be that of a healthy gland; and, because of the obliteration of the vessels from causes named above, attended with far less hæmorrhage.—*Amer. Jour. Med. Sciences.*

